# Basic Occupational Health Services for Agricultural Workers in the South of Iran

**DOI:** 10.29024/aogh.2312

**Published:** 2018-08-31

**Authors:** Ramin Tabibi, Shahram Tarahomi, Seyed Mohammad Ebrahimi, Ali-Asghar Valipour, Sasan Ghorbani-Kalkhajeh, Shokat Tajzadeh, Davood Panahi, Shahrzad Soltani, Kenesh O. Dzhsupov, Maryam Sokooti

**Affiliations:** 1 Abadan School of Medical Sciences, Abadan, IR; 2 International School of Medicine, Bishkek, KG; 3 Milan, IT; 4 Department of Toxicology, School of Pharmacy, Ahvaz Jundishapur University of Medical Sciences, Ahvaz, IR; 5 School of Public Health, Shahid Beheshti University of Medical Sciences, Tehran, IR

## Abstract

The agricultural sector has by far the world’s largest labour force, there are more than one billion workers in this industry worldwide, which contains half of the total world labour force. On the other hand, agriculture is one of the most hazardous occupations, and many workers suffer occupational accidents and ill health each year. Farming and animal breeding are associated with exposure to a wide variety of risk factors, including zoonotic agents, dust, elements of the thermal environment, noise, vibration and chemicals. Although half of the world’s population are economically active and spend at least one third of their time in the workplace, only 15% of the workers have access to basic occupational health services. According to a WHO report, Iran has a well-structured health care system through which basic health care services are available to the entire population, and health indicators in Iran have consistently improved. The agricultural health program in Iran is being carried out in the cities and rural areas, and occupational health services are mainly integrated into the health network. This paper aims to describe the health care system and basic occupational health services (BOHs) available to 5,300 agricultural enterprises with 8,380 employees in the cities of Abadan, Khorramshahr and Shadegan in the Abadan region (Abadan, Khoramshahr and Shadegan districts), in the south of Iran.

## Background

The concept of Basic Occupational Health Services (BOHS) comes from the WHO Alma Ata Declaration from the year 1978: “Primary health care is essential health care based on practical, scientifically sound and socially acceptable methods … It is the first level of contact of individuals, the family and community with the national health system bringing health care as close as possible to where people live and work …” Occupational health services are unequally distributed in the world [1]. This is the fact that more than 80% of workers in the world work and live without having access to occupational health services (OHS) [2].

Farm work is one of the most dangerous occupations in the world. Using heavy machinery, including tractors, harvesters, and others, causes the most injuries and fatalities in agricultural work. In addition to the safety issues, there are health hazards that can result in work-related diseases. The main risks in agriculture are chemical, physical and biological agents, and exposure to these agents can result in immediate and serious consequences as well as chronic or long-term consequences [3]. All around the world, in developed and especially in developing countries, many risk factors threaten the workers in the agricultural sector. These factors can lead to cancer, respiratory diseases, musculoskeletal diseases, poisoning due to the use of pesticides, liver disease, skin diseases, osteoarthritis and injuries resulting from accidents in the workplace [4,5,6,7].

It has to be noted that just 9% of agricultural workers are in industrialized countries. Almost 60% of them are in developing countries. It is the largest sector for female employment, especially in Africa and Asia [8].

Constant use of chemicals causes death and maims of thousands through persistent exposure, accidents or misuse. Agrochemicals can also cause long-term illnesses, reproductive impairments and ongoing harm to the Earth and its resources. According to the Occupational Safety and Health Administration (OSHA), during 2003 and 2011, about 5,816 agricultural workers died from work-related injuries in the US. It was stated that just in 2011, 570 agricultural workers died from injuries due to work. In general, the fatality rate for agricultural workers was seven times higher than it was for workers in the private sector [9].

## Country Profile

Iran, with a population of 81 million, has the second largest economy (after Saudi Arabia) in the Near East and North Africa region. Iran ranks second in the world in natural gas reserves and third in oil reserves. The agriculture and rural sector’s share in the GDP has declined in the last 20 years and yet is the source of income for more than 15 million people in rural areas. One quarter of the rural population is landless and of those who own land, one third are smallholders. Those within this segment of the population often fall below or just within the poverty line and face high underemployment rates.

The Islamic Republic of Iran is located in the Middle East and covers a total area of about 1.75 million km². The country is bordered by Armenia, Azerbaijan, the Caspian Sea and Turkmenistan to the north, Afghanistan and Pakistan to the east, the Gulf of Oman, the Strait of Hormuz and the Persian Gulf to the south, and Iraq and Turkey to the west. It is estimated that 52% of the country is covered with mountains and deserts and about 16% of the country has an elevation of more than 2,000 m above sea level [10,11].

## The state of the agricultural sector in Iran

Iran is among the first countries in the world in history to have agriculture and cultivating. According to United Nations data, 18% of Iranians are engaged in the agricultural industry [12]. Approximately one third of the lands in Iran have agricultural potential, but because of poor soil and inappropriate water distribution in most areas, cultivation in most of the arable land is not practiced in Iran. Only 12% of Iran’s vast area is under agricultural operations (including gardens, vineyards and cultivated lands), but less than one third of the arable land is irrigated, and the remainder is under dry farming. The north and northwest of Iran have fertile soil. According to available information from 231 countries, about one third of them are leading agricultural production, which ranked first to tenth of the world in producing one or more agricultural products. In this regard, Iran has a good position; it has world-class agricultural products. Iran has a world-wide rank of 15 important garden products, in terms of the diversification of garden products. However, the agricultural sector suffers from the lack of mechanization and the use of new agricultural and irrigation equipment. While the number of tractors per 1,000 hectares of land in the world is about 18 tractors, in Iran that is 14 tractors. Also, the value of products per farmer in Iran is about $2,450, which is lower than the Middle East and North Africa, while at the same time higher than the global average.

Iran and the Food and Agriculture Organization of the UN (FAO) have collaborated over several decades for the purpose of promoting the sustainable development of the country’s agriculture and rural sectors. Recently, the FAO has mainly focused on agricultural planning and optimizing capacity development and improving the agricultural productivity of the country [13].

## Health system in Iran

The Ministry of Health and Medical Education (MOHME) is responsible for planning, monitoring and supervision of health-related activities for the public and private sectors in Iran [14]. Primary Health Care (PHC) was established in Iran in 1978 and is known as a national infrastructure for providing health care services. The World Health Organization (WHO) recommends all countries to follow their health program according to the Alma-Ata Declaration of 1978. At present, the Environmental and Occupational Health Center (EOHC) at MOHME is responsible for supervising occupational health programs and activities at a national level. PHC has been organized in order to benefit all its aspects, from family to the hospital [15].

According to a 2006 WHO report Iran has a well-structured health care system in which basic health care services are available to the entire population and guaranteed by the Iranian Constitution. Evidence says that since the early 1980s, health indicators in Iran have consistently improved, to an extent that can be compared with those in developed countries [14,16].

In recent years the provision of occupational health services in PHC has been developed in many parts of Iran. The provision of health care services in Iran includes all manufacturing, industrial, services, developmental and agricultural occupations. Providing agricultural health services to the workforce in Iran by rural health centers, urban health centers, occupational health units (the labor health centre, industries and firms with more than 500 employees) and worker health houses (in industries with less than 500 people) are conducted [17]. However, the coverage of the health care system for the jobs related to agricultural sector, seems weaker than other industries [18].

The agricultural health program in Iran is being carried out in the cities and rural areas with a population of under 50 thousand, as well as in the cities with a population of 50 thousand and over. Occupational health services for agricultural workers in Iran is provided in two forms of occupational health services and occupational medicine.

The cities of Abadan, Khoramshahr and Shadegan are located in the southwest of Iran. In southwestern Iran, jobs in agriculture are mainly in sugar cane and palm farms, vegetables (such as wheat, okra and other vegetables), fish, poultry, livestock and animal husbandry (mostly traditional farms). Also, some workers in this area do fishing (fishing from the Karun River, Arvand, and Persian Gulf).

According to Table 1, there are about 5,300 agricultural enterprises with 8,380 employees in the cities of Abadan, Khorramshahr and Shadegan. The lowest provided health care services are related to agriculture and gardening and animal husbandry, because of difficulty accessing to these workers and the family farms, which are mostly run in a traditional way, with one to three employees. Unfortunately, there is no law that obliges the workers to participate in health education courses and to do job examinations. Workplace inspections and face-to-face training are the only services that can be provided to these workers according to the current rules.

**Table 1:** Agricultural employment and workers’ activities in three main cities (Abadan, Khorramshahr and Shadegan) in Khouzestan province, Iran, 2017.

Type of job	Number of Workshops	Number of employees

Agriculture and gardening	4739	4771
Sugar cultivation industry (large-scale independent farms)	3	2630
Animal husbandry	282	296
Poultry	30	35
Fish/shrimp breeding	40	314
Date packing (family farms)	205	331
Mushroom planting	1	3
**Total**	**5300**	**8380**

It should be noted that because of the low level of education in this sector and probable lack of health beliefs, the training provided by the health inspector at the workplace does not result in significant health improvement in the mentioned workplaces. Other jobs in this table have better situations.

One of the main obstacles in identification of the workers engaged in the agricultural sector (especially traditional fishermen and farmers) for the purpose of providing occupational health services, workers in Abadan, Khoramshahr and Shadegan is access to them. It seems that there is no official source to identify some of the workers, especially farmers who work in the field of fishing and traditional farming in these regions. In other jobs other than fishing and traditional animal husbandries, identifying the status of workers due to the availability of work history of the employees from the Department of Agriculture seems easier. However, the Iranian health system makes every effort to cover workers and farmers with basic occupational health care services.

In the three cities of Abadan, Khoramshahr and Shadegan, periodic occupational health visits are one of the services provided to all the farmers by the Iranian health system. These examinations are conducted annually. Occupational health services in the cities will also include health education, mainly through face-to-face training, and during the inspection of agricultural workshops and group training for training in classes, to measure the work-related risk factors in the agricultural industry and to monitor and control these risks at the workplaces. According to available data from the occupational health unit of the Abadan Health center, in 2016 around 2,700 workers completed occupational health examinations out of a total of 8,000 agricultural workers.

In the occupational health examinations, for each worker or farmer, a specific data form will be filled out by the health staff or physicians. The visit of the workplace will be before or after completing the form which can be filled out by health personnel or by the physician. This form includes personal details, occupational records, assessment of the occupational hazards, medical history of the employed person (history of disease or defect), examinations, lab tests, paraclinical examinations, counseling and referral results, and finally the ultimate recommendations by an occupational physician. Occupational health professionals and general practitioners are trained to complete the form and recognize the specific risks factors of farms and workplaces. During the examination, a physician will investigate the signs and symptoms as well as the general state of the person, and the lab tests will be performed for workers (CBC, UA, FBS, TotalChol, LDL, HDL, TG, BUN, Cr, ALT, AST, ALK.Ph, PSA, HBS Ag, S/E & OB and PPD). In addition, paraclinical tests including optometry, audiometry, spirometry and ECG will be carried out [19].

The most common diseases of farmers in Abadan, Khoramshahr and Shadegan are the musculoskeletal disorders (MSDs). It has to be noted that, in the agricultural farms, monitoring the risk factors of the workplace and risk assessment can be carried out by the authorized private occupational health companies approved by the Ministry of Health. These risk factors include physical agents (radiation, thermal stresses, sound, lighting, vibration, magnetic field, electric field, etc.) or chemical and ergonomic factors of the workplaces. If there are excessive harmful factors in the work environment, the employer has to do the necessary actions to eliminate the risk of the exposure of the worker to the risk factors. The occupational physician, after performing the essential examinations, if necessary, may refer the employee for further diagnosis and treatment to one the specialized and modern hospitals in the city.

Some indices regarding the occupational health activities of Abadan University of Medical Sciences (AUMS) and comparing these with the level of the country are shown in Table 2. These indices are the available statistics on occupational health that are formally presented in Iran. The high number of visited workplaces in Abadan, Khoramshahr and Shadegan cities are due to increased number of health personnel in these areas. It is also shown that there were more workplaces with their own OH units, which is more than the country’s average. A majority of inspected workplaces were industries, services, repair shops and carpenters.

**Table 2:** Some selected indices of the OH unit of AUMS and comparing these with the country in 2016.

No	Index	Percentage	

		AUMS	IRAN

1	Visited workplaces	98.48	65
2	Visited workers through OH surveillance programs	54.28	NA
3	Workers who are covered by OH Services for measuring and controlling risk factors at work place	68.05	NA
4	Workplaces with occupational health units	42.50	33.0
5	Workers at risk of high levels of noise	8.62	18.0
6	Workers with exposure to chemical hazards	12.29	NA
7	Workers with inappropriate posture during working hours	16.25	28.0
8	Workers with hearing loss problems	7.83	5.7

## Discussion and Conclusion

Out of the 3 billion workers in the world, around 80% do not have access to occupational health services (OHS). The Basic Occupational Health Services (BOHS) are indeed an application of the Primary Health Care policy in the sector of occupational health. BOHs are vital for the protection of people’s health at work, for promotion of health, well-being and work ability, as well as for prevention of ill health and accidents. Some of the main principles that are applied within BOHS include availability to all workforces, adaptation to local conditions and addressing the local needs [20].

At present, there are few countries like Iran that are reforming their health systems based on PHC to improve the quality of their services. One of the main objectives of integrating occupational health services in PHC in Iran, as mentioned at the Hague international conference, was providing access to basic health services and prevention of occupational and work-related diseases and injuries for all workers at the workplace [17].

According to the definition of the International Labor Office (ILO), “Occupational health surveillance is the ongoing systematic collection, analysis, interpretation and dissemination of data for the purpose of prevention [21].” With regards to the underreporting of occupational diseases in an agricultural setting, there is a need for the basic occupational health services to farm workers in southwest Iran, and the execution of health surveillance activities for farm workers depends upon the possibility of a system that can reach the workers at their workplaces. The OH and health surveillance program is based on physical examination, chemical risks, electrocardiography, hearing and lung function examinations, and using specific tests addressing risks like vibration, physical overload, chemicals, biological agents and allergens [22].

The overall goal of occupational health programmes for farmers is the improvement of the level of health of farmers in the agricultural sector. In Abadan, the agricultural health plan was launched to maintain health promotion among farmers. This program is well supported by MOHME, Abadan University of Medical Sciences and the private sector (Arvand free zone organization).

There are also some obstacles for the occupational health activities and health education for the farmers, including a lower level of farmers’ information regarding the adverse effects of occupational hazards, unwillingness of farmers and animal breeders to attend training sessions or medical visits and the lower level of education among farmers.

It was shown that proper documentation of health surveillance and OH services findings will provide a safe basis for employers, employees and occupational safety and health specialists in case of legal claims. These data can also provide a starting point for further scientific researches.
